# Comparing health outcomes in SHARE with other European surveys

**DOI:** 10.1186/1753-6561-7-S4-S2

**Published:** 2013-08-23

**Authors:** Simone Croezen, Mauricio Avendano, Alex Burdorf, Frank J van Lenthe

**Affiliations:** 1 Department of Public Health, Erasmus MC, Rotterdam, the Netherlands; 2 LSE Health, London School of Economics and Political Science, London, UK

## 

In the Survey of Health, Ageing and Retirement in Europe (SHARE) (http://www.share-project.org), health measures were constructed based on a careful consultation process of health experts and previous health surveys. However, there have been limited efforts to examine the comparability of health data in SHARE. Therefore, we aim to compare self-reports of health based on incidence and prevalence of health outcomes in SHARE to estimates from parallel national and trans-national health surveys.

Prevalence of health outcomes in SHARE measured at baseline (2004/2005) were compared to prevalence estimates from the European Health Interview Survey (EHIS) (2006-2009), national Health Interview Surveys (HIS) (1997-2003), the European Social Survey (ESS) (2004/2005), the EU Statistics on Income and Living Conditions (EU-SILC) (2005) and the disability ad hoc module added to the European Union Labour Force Survey (EU-LFS-disability) (2002). Components of each of these surveys provide an element of comparison for a particular health dimension in SHARE. Across countries, agreement between surveys was described for self-perceived health, long-standing illness, global activity limitations, diabetes, hypertension, asthma, lung diseases, depression and overweight by age group, gender and educational level.

First results show substantial variation in the comparability of health prevalence assessed by SHARE with other health surveys, depending on the health indicator of interest. For instance, a good agreement with SHARE was found for self-reported diagnosed diseases assessed by EHIS and for overweight based on self-reported weight and height by EHIS and HIS. On the other hand, for self-perceived health, the agreement between SHARE and other surveys was poor. Furthermore, agreement varied across countries, making country-specific comparisons between surveys less reliable. Lack of agreement between surveys is studied in more detail concerning systematic differences in survey design, sampling strategies, responses and assessment modes. Investigating time trends will evaluate whether differences in prevalence of health indicators between surveys will also affect agreement among trends over time in prevalence of health measures across age, gender and education. These additional analyses will complete this first assessment of the quality of SHARE data on health. Besides, this research will highlight the overlap and complementarity between SHARE and other national and trans-national health surveys. By recognising health measures that could be harmonised between surveys and measures that are more exclusive, we could exploit the opportunities offered by having multiple measures of health in different surveys.

